# Diaphragm-Directed Strategies for Prolonged Air Leak Prevention After Lung Resection: A Narrative Review of the Residual Pleural Space Paradigm

**DOI:** 10.3390/jcm15187081

**Published:** 2026-09-12

**Authors:** Ruoran Xia, Evgeniy Tarabrin, Sergey Muraviev, Milena Ivanova, Zelimkhan Berikkhanov

**Affiliations:** Department of Hospital Surgery No. 2, Sechenov University, 119991 Moscow, Russia; li4781955@gmail.com (R.X.); tarabrin_e_a@staff.sechenov.ru (E.T.); muravev_s_yu@staff.sechenov.ru (S.M.); ivanova_m_yu_1@staff.sechenov.ru (M.I.)

**Keywords:** prolonged air leak, residual pleural space, lung resection, intrapleural pressure, diaphragm, phrenic nerve, botulinum toxin

## Abstract

**Background/Objectives:** Prolonged air leak (PAL) remains one of the most common and costly complications after lung resection. A growing body of evidence implicates the residual pleural space (RPS) as a central pathogenetic link between recognized risk factors and persistence of the alveolopleural fistula. This review synthesizes evidence on RPS pathogenesis and compares six interventions that reduce RPS by acting on the diaphragm for PAL prevention. **Methods:** A narrative literature search was performed in PubMed, Google Scholar, Web of Science, eLibrary.ru, and China National Knowledge Infrastructure (CNKI), covering clinical and experimental studies, systematic reviews, and classic physiological work relevant to intrapleural pressure (IPP), RPS, and diaphragmatic control. **Results:** RPS and PAL are reciprocally linked, and major, upper-lobe, and right-sided resection independently predict both. RPS is accompanied by excess negative IPP, reduced lung compliance, and disproportionate wall stress at the resection margin. Six methods for reducing RPS volume through diaphragmatic control are described. **Conclusions:** RPS is the mechanistic link connecting established PAL risk factors to fistula persistence. Among the six methods, chemical denervation with botulinum toxin type A (BTX-A) is, in principle, the only method combining full reversibility, dose-dependent control, and no need for dedicated equipment. However, this conclusion rests on a single pilot animal study in a lower-lobectomy model, and translating it to upper-lobe, low-reserve patients, who carry the greatest PAL burden, requires dedicated dose-ranging and safety work before a clinical trial can be considered.

## 1. Introduction

Prolonged air leak (PAL) is among the leading causes of extended hospitalization in thoracic surgery. A variety of studies have shown that the incidence of PAL (under different definitions) varies substantially by resection type and population, from 5.4% to 26.3% [1,2,3,4,5]. Under the joint consensus statement of the European Society of Thoracic Surgeons (ESTS), the American Association for Thoracic Surgery (AATS), the Society of Thoracic Surgeons (STS), and the General Thoracic Surgery Club (GTSC), PAL is defined as an air leak through the chest drain persisting beyond five postoperative days [6].

Air leak resulting directly from mechanical injury to the parenchyma or to the staple line usually resolves spontaneously within a short period of time [7,8]. A more severe variant exists, however, in which a residual pleural space (RPS) forming during the postoperative period subjects the resection margin to cyclical respiratory loading [9,10]. This loading appears to drive the transition from a transient leak to clinically significant PAL [9,10,11,12]. Because diaphragmatic respiratory excursion is the principal source of this cyclical load on the resection zone, temporary and controlled restriction of diaphragmatic movement has been proposed as a pathogenetically grounded preventive strategy [13,14].

This review compares six existing strategies that reduce RPS volume by altering the position or function of the diaphragm: pneumoperitoneum, phrenic nerve crush (phrenicotripsy), catheter-based anesthetic block, phrenic nerve infiltration, cryoneurolysis, and chemical denervation with botulinum toxin type A (BTX-A) [13,14,15,16,17,18,19,20,21]. We refer to them collectively as diaphragm intervention methods. They act at different levels of the neuromuscular pathway, from indirect volume displacement to direct nerve block to neuromuscular junction blockade, and differ in site of action, reversibility, postoperative adjustability, and equipment requirements. BTX-A is the most recently proposed of the six and, as later sections detail, the least clinically tested.

The aim of this review is to elucidate the role of residual pleural space functions as a common pathway linking otherwise disparate PAL risk factors, and to compare, on that basis, the diaphragm intervention methods proposed to interrupt this pathway, with particular attention to the pharmacological rationale and evidence base for botulinum toxin type A. Prior work in this area has generally treated these strategies individually or as a secondary mention within broader discussions of air-leak prevention. The contribution here is to place them within one explicit pathogenetic model and compare them systematically against it.

## 2. Materials and Methods

A narrative literature search was conducted in PubMed/MEDLINE, Scopus (Elsevier), Web of Science (Clarivate), eLIBRARY.RU, and China National Knowledge Infrastructure (CNKI). Search terms were combined in different Boolean combinations (AND/OR) across four concept groups: prolonged air leak and its synonyms (persistent air leak, alveolopleural fistula, bronchopleural fistula); the residual pleural space and its synonyms (residual postoperative pleural space, postresectional pleural space); intrapleural pressure and pleural mechanics; and the individual diaphragm-control methods considered here, searched by name (pneumoperitoneum, phrenic nerve crush, phrenicotripsy, phrenic nerve block, cryoneurolysis) and by agent (botulinum toxin).

No fixed publication-date limit was applied. Classic physiological studies from the 1970s through the 1990s were deliberately retained wherever they remain the primary source for a specific quantitative claim used in the biomechanical model developed here, for example, the vertical gradient of intrapleural pressure, alongside contemporary clinical and experimental literature. No language restrictions were applied. Non-English publications were included to incorporate regional clinical data unavailable in international databases.

Eligible evidence types included randomized controlled trials, prospective and retrospective cohort studies, case series and case reports, ex vivo and animal experimental studies, and classic physiological research. This range reflects the review’s purpose, which is mechanistic synthesis rather than aggregation of a single clinical outcome, so foundational physiological work sits alongside clinical trial data as primary evidence for different parts of the argument. As a narrative rather than a systematic review, no formal risk-of-bias assessment, dual independent screening, or PRISMA-type flow diagram was applied, and no review protocol was registered. Source selection reflects relevance to the pathogenetic model and the method comparison developed here, consistent with standard practice for narrative reviews of this kind. Reference lists of key articles were hand-searched for additional sources.

## 3. Results

### 3.1. Risk Factors Shared by Residual Pleural Space and Prolonged Air Leak

RPS is the portion of the pleural cavity not occupied by re-expanded lung tissue after resection, identified radiographically or by computed tomography as an air-containing space between the visceral and parietal pleura [22,23]. RPS is usually visible on imaging within the first one to two postoperative days, while PAL, by definition, cannot be diagnosed until an air leak has persisted for five days, so RPS formation generally precedes PAL diagnosis itself and can be assessed independently of it [7,22,23,24,25]. RPS rates vary widely across series, depending on the diagnostic criteria applied and the imaging modality used. Solak et al. followed patients with RPS present on the first postoperative day; the space was reabsorbed by week 12 in 75.8% of these patients, persisted in 10.4%, and was associated with related complications in 13.7% [22]. Misthos et al., in 966 patients, recorded RPS in 9.5% of operated patients, occurring significantly more often after upper lobectomy, in malignant disease, with apical location, and on the right side [23]. In a more recent series, Vita et al. found RPS in 325 of 492 patients (66.1%) after uniportal video-assisted thoracoscopic surgery (VATS) lobectomy, measured by the Collins method; mean RPS volume was 15.46 ± 8.59% versus 4.2% in patients without RPS. An RPS volume above 10.5% was associated with a higher risk of postoperative air leak (AUC 0.69) [5]. A different multivariable model looked at RPS presence on its own, and in that model PAL was one of four independent predictors, along with low body mass index, active smoking, and right-sided surgery [5]. The same cohort’s own PAL rate was 9.8%, a direct within-study comparison of the two outcomes that most other sources in this review cannot offer [5]. A further cohort measured RPS in a different population. Rocha Jr et al. followed 135 patients undergoing lung resection for infectious disease, most commonly tuberculosis, and found RPS on the fourth postoperative chest X-ray in 76 patients, 58% of the cohort [26]. Patients with RPS in this cohort faced a higher risk of infectious pleuropulmonary complications (RR = 1.47; 95% CI: 1.12–1.92; *p* = 0.01) [26]. This population differs from the lobectomy and segmentectomy cohorts shown above; most of them were operated on for cancer or nodules, so its incidence figure is not directly comparable to the others [26].

The extent of resected lung tissue is among the most reproducible predictors of PAL across the literature. Large-volume anatomical resections, lobectomy and bilobectomy, are consistently associated with a higher rate of PAL than sublobar resections, segmentectomy and wedge resection. Pischik et al. reported that lobectomy and bilobectomy lead to PAL significantly more often than segmentectomy and wedge resection, and identified staple-line length as an independent predictor in its own right [27]. In a retrospective cohort of 1060 patients, Kim et al. similarly reported that major resection was an independent predictor of PAL, with an adjusted OR of 1.80 (95% CI 1.56–2.08; *p* < 0.001) after adjustment for age, sex, body mass index, pulmonary function, surgical approach, and duration of one-lung ventilation [28]. The same group also studied an intraoperative measure called ventilatory leak, defined as the discrepancy between inspiratory and expiratory tidal volume once two-lung ventilation resumed, which independently predicted PAL [28]. Unlike most PAL risk factors, which are known only before surgery or only in retrospect, ventilatory leak can be measured in the operating room while it is still forming [28]. Several other cohort studies have measured PAL in recent large unselected surgical populations. One of these large-scale studies drew on the French national thoracic database, 24,113 patients in the development cohort alone, and recorded PAL in 6.9% of that population [29]. This study defined PAL as an air leak beyond seven days, a broader window than most other studies, which likely explains why its incidence figure sits toward the lower end of the range. The resulting model, known as the index of prolonged air leak (IPAL), remains one of the most widely cited PAL prediction tools in the field.

Among lobectomies, the highest reported risk of PAL is associated with upper and right-sided resections. In a multivariable analysis, Petrella et al. found that a right-sided operation is an independent risk factor for PAL (OR 4.46; 95% CI 1.83–10.9; *p* = 0.0010) [30]. Vita et al., in the RPS-focused series described above, confirmed right-sided surgery and upper lobectomy as predictors of RPS rather than PAL directly, which is consistent with the two outcomes sharing risk factors because they share a mechanism [5]. Billé et al. reported a dose-dependent relationship between early leak volume and subsequent PAL: 75% of patients with a leak volume of 180 mL/min or more on the second postoperative day went on to develop PAL, against only 3.1% of those with an air leak <180 mL/min (*p* = 0.0001) [31]. Low FEV_1_, low diffusing capacity, and computed tomography (CT)-confirmed emphysema are independently reported to raise the PAL risk associated with larger resections further [7,30,31]. A different kind of risk factor emerges from Ma et al., who studied a retrospective cohort of 110 patients undergoing video-assisted thoracoscopic resection. PAL occurred in 26.3% of this cohort. Their multivariable model identified two independent predictors, chronic obstructive pulmonary disease (OR = 9.023, *p* = 0.003) and pleural adhesions (OR = 3.404, *p* = 0.013) [4].

The wide variation in reported RPS incidence across studies is summarized in Table 1 to illustrate how differences in diagnostic criteria, measurement methods, assessment timing, and operated populations can explain the observed heterogeneity. Addressing this heterogeneity requires further research focused on standardized RPS definition and prospective volumetric assessment of the residual space under controlled postoperative conditions. RPS and PAL rows are interleaved by year and not grouped by outcome, so risk factors shared between them can be read across outcome types.

### 3.2. Physiological and Biomechanical Measurements Associated with the Residual Pleural Space

Under normal conditions, intrapleural pressure (IPP) varies with lung height. This pattern was first described in classic animal experiments. In the head-up dog, pleural surface pressure at the apex is approximately −8 cmH_2_O, while the vertical pressure gradient ranges from 0.2 to 0.9 cmH_2_O/cm depending on the measurement technique used [34,35]. A subsequent study by the same group found that inactivation of the diaphragm altered the pattern of IPP distribution across the height of the pleural cavity [36]. A comparable gradient, 0.24 ± 0.02 cmH_2_O/cm, has since been demonstrated using magnetic resonance imaging-based estimates of gravitational stress in the supine position [37]. This gradient has clinical significance. A highly significant, robust correlation exists between the entry height of the coaxial needle during lung biopsy and the subsequent occurrence of pneumothorax [38]. Elevating the entry point from the lowest to the highest position across the total lung height increases the relative risk of pneumothorax by up to 110-fold [38]. Direct measurements in patients after pulmonary lobectomy provide the clinically relevant range. In a prospective study of 203 patients, Refai et al. recorded IPP in the hour before chest tube removal and reported mean maximum, minimum, and differential pressures of −6.1, −19.5, and 13.3 cmH_2_O, respectively [39]. Mean pressures were similar across lobectomy types, −11 to −13 cmH_2_O, with the exception of right upper bilobectomy, at −20 cmH_2_O [39]. Bok et al. similarly measured a mean IPP of −11.2 cmH_2_O during quiet breathing after lobectomy when re-expansion was incomplete, with the mean pressure reaching −18.9 cmH_2_O during forced respiration [40].

Compliance, the change in lung volume per unit change in pressure, necessarily falls after resection because there is less lung to expand. This is a geometrically inevitable consequence. The findings of Salito et al. provide evidence for this. In an experimental rabbit model, reduced compliance and an increased risk of overdistension followed hydrothorax and lobar resection once a pleural drain was placed [41]. In a later study, this time a clinical series undergoing VATS resection, the same group quantified a comparable pattern. The measured postoperative loss of compliance exceeded that predicted from the mass of resected tissue alone by 10–15%; compliance correlated with resected-tissue mass (r^2^ = 0.68), and the rate of pleural fluid drainage was inversely related to both remaining-lung mass and postoperative compliance [42]. Given the sample size (*n* = 11), this clinical finding is best regarded as hypothesis-generating. Salito et al. demonstrated a mechanism: this additional loss of compliance reflects mechanical overdistension of the remaining lung during re-expansion, which in turn disturbs pleuropulmonary fluid balance [42].

**Figure 1:**
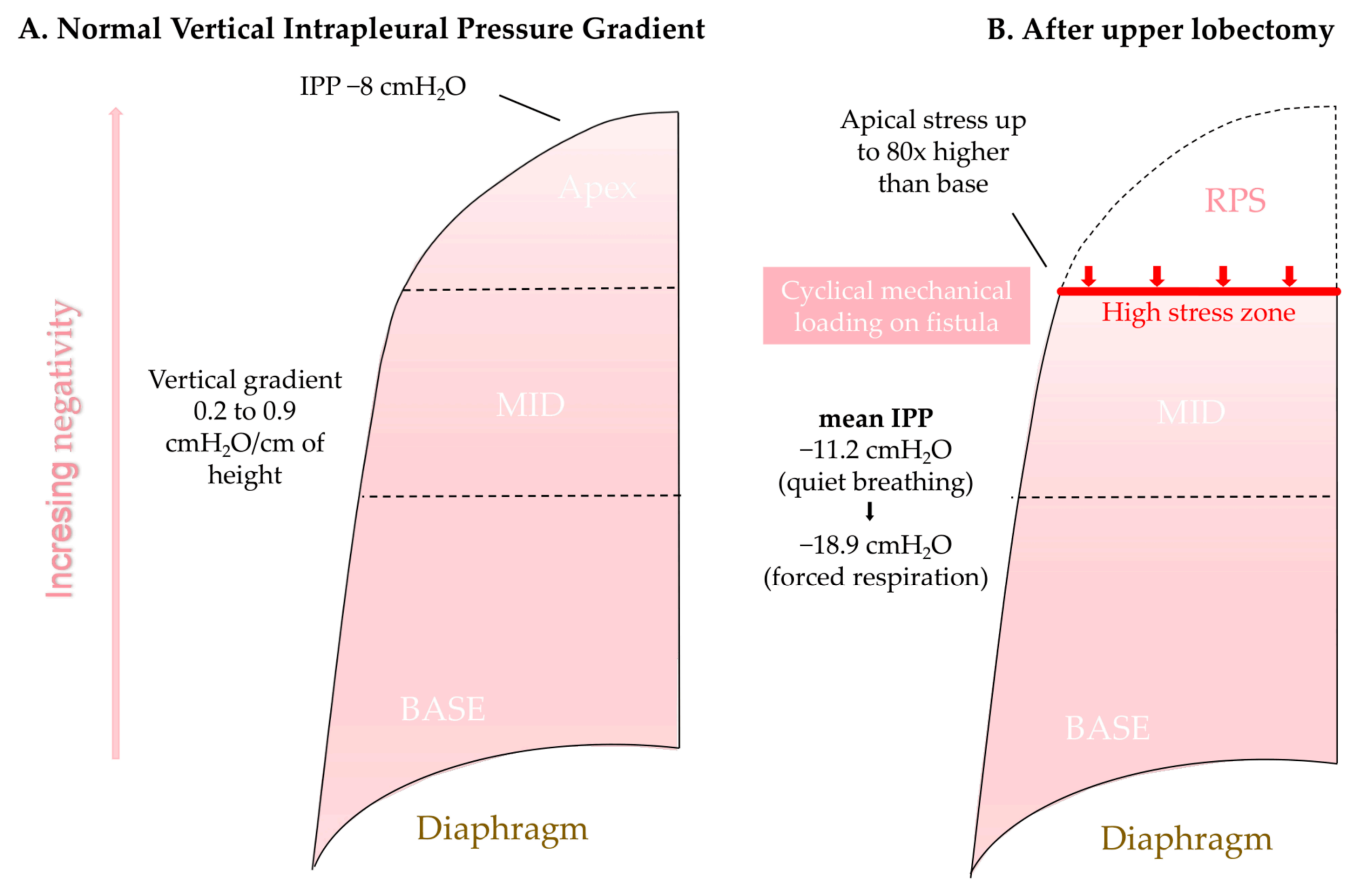
IPP gradient under normal conditions (Panel (**A**)) and after upper pulmonary lobectomy (Panel (**B**)). Panel (**A**): The apex sustains the most negative IPP and the greatest transpulmonary pressure, decreasing progressively toward the base. This gradient direction is well established, driven by gravity acting on lung tissue and pleural fluid. Panel (**B**): After upper lobectomy, RPS forms above the resection margin, and IPP subsequently becomes more negative. Finite-element modeling demonstrates an 80-fold amplification of apical versus basal wall stress after upper lobectomy. Cyclical diaphragmatic excursion delivers this load to the alveolopleural fistula at the resection margin with every breath, sustaining the RPS and preventing mesothelial repair.

Using finite-element modeling, Casha et al. reported that after upper lobectomy the stress at the apex of the lower lobe can exceed that in the basal regions of an intact lung by as much as 80-fold [43]. A related biomechanical analysis by the same group, framed around the Young–Laplace relationship, found apical pleural stress to be as much as 20 times higher than in the rest of the lung in chests of low thoracic index, a shape characteristic of patients with spontaneous pneumothorax [44]. Petrella et al. described a radiological “balloon-like” sign, a rounded lung contour in the absence of active air leak against a conical contour when a leak persists, and proposed it as a way of distinguishing active leak from isolated RPS on imaging [24]. Figure 1 traces these relationships, from the normal IPP gradient to the mechanical consequences of lobectomy.

### 3.3. Diaphragm Interventions for Reducing the Residual Pleural Space

Pneumoperitoneum. In a randomized trial of 30 patients undergoing lower lobectomy or bilobectomy, Okur et al. applied intraoperative pneumoperitoneum through a catheter passed via the diaphragm from the pleural cavity [18]. They reported shorter drainage time (3.5 versus 4.9 days in controls), shorter hospital stay (4.7 versus 5.8 days), and fewer cases of RPS (1 versus 8 patients), with no increase in other complications [18]. Subsequent prospective series report similar safety after lobectomy and bilobectomy [45,46]. In a larger series combining pneumoperitoneum with autologous blood patch in 39 patients with established RPS and air leak after major resection, Korasidis et al. reported obliteration of the pleural space in all patients within 96 h and cessation of air leak within 144 h, with a mean postoperative stay of 8 days and no recurrence at four-month follow-up [25]. The same group notes that diaphragmatic elevation is one of several physiological mechanisms, alongside mediastinal shift and expansion of the residual lung, by which the body itself partially compensates for a residual pleural space [25], which is consistent with treating diaphragmatic elevation as an amplification of an existing compensatory mechanism rather than an artificial intervention.

Phrenic nerve crush (phrenicotripsy). Iverson et al. reported that surgical crush of the phrenic nerve reduces diaphragmatic function and can produce permanent paralysis [47]. Persistent unilateral diaphragmatic paralysis is reported to produce a fixed restrictive pattern with a marked reduction in FVC (forced vital capacity) and FEV_1_ (forced expiratory volume in 1 s), and surgical plication performed years after such paralysis is reported to achieve only partial functional recovery [48].

Catheter-based anesthetic block. Clavero et al. described continuous para-phrenic bupivacaine infusion providing controllable diaphragmatic paralysis for RPS [17]. Carboni et al., in six patients after lobectomy and bilobectomy, confirmed fluoroscopically that infusion of 1% lidocaine through a perifrenic catheter produced diaphragmatic paralysis, with function returning immediately on cessation of the infusion [16]. Patella et al. reported that in 10 patients with established PAL, injection of 0.2% ropivacaine through a catheter (Contiplex C, BBraun, Melsungen, Germany) positioned near the phrenic nerve at the C6 level stopped the leak after a mean of 3.0 ± 1.16 days [13].

Phrenic nerve infiltration. Trabalza Marinucci et al., in the only trial of its kind to date, infiltrated the perineural fat overlying the pericardium with ropivacaine (10 mg/mL) in 22 of 65 randomized patients at high risk of PAL undergoing lobectomy or anatomical segmentectomy [19]. The infiltration group showed a significant rise in hemidiaphragmatic position (*p* = 0.006), better lung re-expansion (*p* < 0.005), and lower pain intensity at 24 and 72 h (*p* = 0.004) [19]. Chest tube duration was shorter (10.18 ± 3.45 versus 15.84 ± 8.97 days; *p* < 0.005), and PAL was less frequent (18% versus 84.2%; *p* < 0.005) [19]. No procedure-related complications were recorded, and the trial did not report the duration of the resulting paralysis.

Cryoneurolysis. Pan et al., in a randomized trial of 207 patients undergoing lobectomy or bilobectomy with systematic mediastinal lymph node dissection, reported that intraoperative cryoneurolysis of the phrenic nerve using a cryoprobe (2 mm JT-1, Kooland, Beijing, China) shortened drainage time (3.2 ± 0.2 versus 4.3 ± 0.3 days; *p* < 0.01) [14]. Meng et al., in a later randomized trial of 108 patients undergoing video-assisted thoracoscopic lobectomy, reported that cryoneurolysis with a cryoprobe (K320, Kooland, Beijing, China) reduced the risk of progression from an intraoperative leak to PAL in the high-risk subgroup (*p* < 0.001). The overall PAL rate did not differ significantly between groups (20.4% versus 14.8%; *p* > 0.05) [15].

Chemical denervation (BTX-A). BTX-A blocks acetylcholine release at the neuromuscular junction, producing reversible flaccid paralysis of the injected muscle [49,50]. In the only experiment testing this agent for this indication, Kaya et al. randomized twelve rabbits after right lower lobectomy to receive either 10 units of BTX-A (BOTOX^®^, Allergan Pharmaceutical Ltd., Westport, Ireland) in the hemidiaphragm (*n* = 6) or saline (*n* = 6). By day 7, the diaphragmatic dome had risen 7.0 ± 2.5 mm in the BTX-A group against 1.3 ± 1.2 mm in controls (*p* = 0.0035), RPS had resolved in all treated animals, and diaphragmatic mobility recovered fluoroscopically at 8–12 weeks, with no respiratory distress in either group [20].

Table 2 summarizes the comparative characteristics of the six methods mentioned above for reducing the residual pleural space.

## 4. Discussion

### 4.1. The Residual Pleural Space as a Pathogenetic Mechanism

The incidence, risk-factor, and pressure data reported above converge on a single pathogenetic account, built from three mutually reinforcing mechanisms.

The first mechanism is blockade of biological repair at the defect. Healing of an alveolopleural fistula requires direct contact between the visceral and parietal pleura, at which mesothelial cells release fibronectin, collagen, and growth factors that initiate the repair cascade [52,53]. When a residual space keeps the two surfaces apart, this contact cannot occur, and the defect stays open, kept patent by the ongoing passage of air through it [52,53]. This is consistent with the clinical observation that pleural adhesions, which hinder both intraoperative visualization and later full lung re-expansion, are independently associated with PAL risk [54]. Whatever else adhesions do, their presence marks patients in whom pleural apposition, and hence repair, is already compromised. Malnutrition and corticosteroid use act on this same repair sequence, since both are established inhibitors of the fibroblast proliferation and collagen deposition this cascade depends on [55,56]. In a patient with hypoalbuminemia or recent high-dose steroid exposure, the mechanical apposition a diaphragm intervention provides may hold the pleural surfaces together without the biological machinery being able to use that apposition to seal the defect. In the Pischik et al. cohort, hypoproteinaemia was an independent PAL predictor [27], consistent with this parallel biological pathway. For such patients, nutritional optimization and minimization of perioperative corticosteroid exposure are priorities that diaphragm intervention cannot replace.

The second mechanism is the excess negative IPP documented above. That pressure is not simply a marker of incomplete re-expansion; it is better read as a measurable trace of the volume deficit the remaining lung is being asked to fill, and every breath converts part of that deficit into mechanical load on the staple line. Experimental work on the isolated lung supports this reading: re-expanding an operated lung of reduced compliance requires pressures well above the physiological range, and exceeding those pressures causes overdistension of the parenchyma, fragmentation of the extracellular matrix, and increased permeability of the alveolopleural membranes [52,57]. The Salito group’s finding that measured compliance loss exceeds what resected-tissue mass alone would predict is consistent with an active mechanical process layered on top of simple loss of lung volume, though, as noted above, the specific causal link the group proposes between mechanical stress and fluid-balance disturbance remains their own stated hypothesis and is not yet a demonstrated mechanism [42].

Combining these data reveals that an intervention method built entirely around evacuating the pleural space as completely and as quickly as possible may itself impede the repair process that PAL prevention is trying to protect. Miserocchi et al. also proposed this view, arguing that pursuing immediate, maximal pleural apposition through aggressive suction works against the operated lung’s own physiology, and some free air should deliberately be left in the pleural cavity, so that transpulmonary pressure stays close to the preoperative level [52].

The third mechanism is cyclical mechanical loading from diaphragmatic excursion, which supplies 60–75% of the tidal volume of quiet inspiration [52,58,59] and is therefore the largest single contributor to the pressure swings described above. D’Angelo et al. found that diaphragmatic contraction, pleural pressure fell more near the diaphragmatic surface than at higher intercostal levels, though this pattern was not clearly present during quiet spontaneous breathing and emerged mainly under direct phrenic stimulation [60]. A related study from the same group found that removing the diaphragm also altered the vertical distribution of pleural pressure [36]. Chest wall shape and lung weight contribute to this same gradient. However, no study has directly compared the relative contributions of these factors within a single multivariable model. The practical case for targeting the diaphragm rests on accessibility; the diaphragm can be modified by plication, pacing, or pharmacological weakening in ways the chest wall and lung parenchyma cannot. The biomechanical basis of the described processes is a simplified model of the Young–Laplace law. Since the pressure required to maintain the alveolus in an expanded state is inversely proportional to its radius, smaller alveoli experience a disproportionately larger transmural gradient [44]. Strictly speaking, this relationship applies to spherical interfaces, whereas real alveoli have a polyhedral shape, and the relationship is relevant only for the small curved regions at the intersections of alveolar walls [61]. Nevertheless, this anatomical limitation does not undermine the equation’s use here, as it serves to illustrate a directional principle that a smaller radius of curvature lowers transmural pressure, without being relied upon for quantitative prediction. The finite-element estimate from Casha et al. offers firmer support for the apical-stress claim made here, since it models the lung’s actual geometry computationally instead of assuming a single sphere: apical wall stress can exceed basal stress by up to 80-fold after upper lobectomy, precisely where PAL risk is highest [43,44]. The same relationship runs in the direction relevant to prevention: reducing RPS volume increases the radius of curvature of the remaining lung surface and, with it, the mechanical stress at the defect. Petrella et al.’s “balloon-like” sign is best understood as the radiological signature of this same equilibrium [24]. A rounded lung contour reflects a defect under low, closing-favorable tension, while a conical contour reflects a defect still being reopened by ongoing airflow.

**Figure 2:**
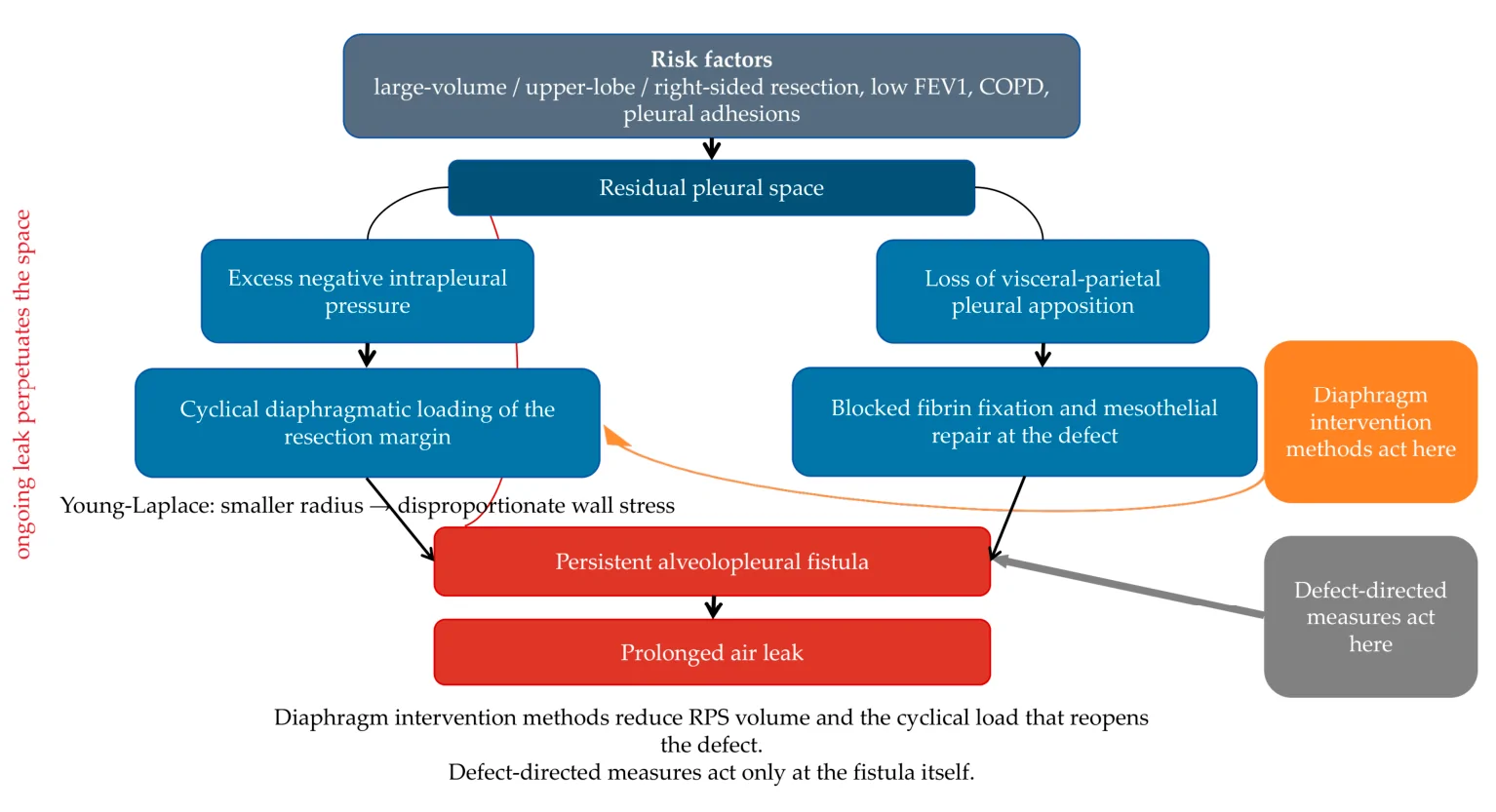
The role of the residual pleural space in the self-sustaining cycle of prolonged air leak. Lung resection in the presence of risk factors (1) creates RPS (2), which branches into excess negative IPP and loss of pleural apposition (3). These converge, through cyclical diaphragmatic loading and blocked fibrin fixation, respectively, on a persistent alveolopleural fistula (4), which in turn keeps the space open, closing the loop. Diaphragm interventions act upstream. Defect-directed measures act only at the fistula itself.

These three mechanisms describe a self-sustaining cycle, as illustrated in Figure 2: RPS → excess negative IPP and loss of pleural apposition → blocked biological repair and cyclical reopening by the diaphragm → sustained air leak. Simply using the defect-directed repair cannot break this vicious cycle. It requires pairing that repair with a reduction in RPS volume large enough to bring wall stress at the defect back into a range where fibrin can fix and repair can proceed [13,18,62,63].

The pathogenetic model developed here receives its most direct empirical support from Vita et al., who measured RPS using the Collins method in 492 patients. RPS exceeding 10.5% was significantly associated with subsequent PAL (AUC 0.69, sensitivity 69%, specificity 54%, *p* < 0.001), and, in a separate multivariable model of the same population, prolonged air leak was itself among four independent predictors of RPS, alongside body mass index (OR 0.95, *p* = 0.015), active smoking (OR 1.64, *p* = 0.044), and right-sided surgery (OR 1.83, *p* = 0.003), with PAL carrying the strongest association of the four (OR 3.89, *p* = 0.003) [5]. Because this study measured RPS as an explicit variable, instead of inferring it, these two findings give the proposed cycle a direct empirical anchor: RPS predicts PAL, and PAL predicts RPS, in two distinct analyses of the same population. As with any retrospective association, this does not establish which came first. PAL status and RPS extent are typically assessed close together in time, often from the same late chest radiograph, so the finding is compatible with genuine bidirectional causation but cannot exclude both measurements simply reflecting one underlying problem recorded twice.

### 4.2. Limits of Defect-Directed Measures

Sealants, staple-line buttressing, pleurodesis, and endobronchial valves all act at the site of the defect itself. None of them alters RPS volume or the cyclical diaphragmatic load described above [62,63]. A sealant applied to a defect that continues to sit inside a large, negatively pressured, cyclically loaded residual space is being asked to hold against exactly the mechanical conditions least favorable to adhesive or biological bond. That framing is consistent with the mixed and generally modest effect sizes reported for sealants and valves in the wider literature [45,63], and it suggests that the ceiling on what defect-directed measures alone can achieve is set by the mechanical environment they are deployed into, not by the properties of the material itself.

This review deliberately concentrates on diaphragm intervention methods and does not examine sealants, pleurodesis, or endobronchial valves in the same depth, because these act on a different link in the pathogenic chain: the parenchymal defect itself, rather than the residual space that keeps reopening it. In practice, the two classes of intervention are not competitors. Intraoperative reinforcement of the staple line removes the immediate source of the leak, while diaphragmatic elevation removes the mechanical conditions that would otherwise sustain any remaining microdefect. Currently, there are no prospective studies that have combined defect sealing with controlled diaphragmatic elevation in a single protocol. This combination is an independent and underexplored line of research in its own right, particularly for patients who carry risk factors from both categories at once. This same distinction is visible in recent advances in the monitoring and conservative management of established PAL. Digital thoracic drainage systems now quantify air-leak flow continuously and objectively, removing the observer variability inherent in conventional bubble-chamber assessment and enabling earlier, criteria-driven decisions on drain removal [63]. Endobronchial valve placement has matured into a bronchoscopic option for patients in whom surgical re-exploration carries prohibitive risk, with published series demonstrating air-leak cessation in the majority of appropriately selected patients [64]. The systematic review provides a contemporary synthesis of intraoperative prevention and conservative management options. However, all of these approaches address the defect itself, and none reduces the residual pleural space or the cyclical diaphragmatic loading that prevents fistula closure. The combination of effective defect sealing with diaphragm-directed space reduction remains the specific research gap.

### 4.3. Comparative Appraisal of Diaphragm Interventions

No head-to-head trial has compared the six methods described here against one another. The comparison that follows is therefore theoretical, built from the pharmacological and technical properties of each method rather than from direct clinical data, and should be read with that limitation in mind.

None of the six methods combines single intraoperative administration, full reversibility, dose-dependent postoperative control, and independence from continuous additional equipment. Each fails on a different axis.

Pneumoperitoneum is supported by the most direct trial evidence [18,25,45,46]. Its site of action, elevation of the diaphragmatic dome from below, matches the basal geometry of lower- and middle-lobe RPS more naturally than it matches the apical RPS that predominates after upper lobectomy, the higher-risk resection identified above. Once the gas is introduced, its resorption rate, rather than the clinical course, sets the duration of effect, and the periodic diaphragmatic contractions that supply the third mechanism above continue throughout. A clinically important parameter absent from all available trial reports is the optimal intra-abdominal pressure (IAP). Published technique descriptions of pneumoperitoneum for RPS reduction target approximately 9 to 10 mmHg [65], well below the 12 to 15 mmHg used in standard laparoscopic surgery [66]. At laparoscopic pressures, elevated IAP reduces respiratory compliance, raises arterial CO_2_ tension, increases peak inspiratory airway pressure, and impairs venous return [67], effects compounded by the already-reduced pulmonary compliance of the postresection lung. The lower pressure range adopted for RPS reduction is consistent with a purely space-filling goal and plausibly explains the absence of ventilatory complications in the published series. No dedicated dose-finding study has established a precise threshold above which respiratory risk outweighs benefit in this thoracic context, and the available trial reports do not specify the IAP used. Defining this threshold is a prerequisite for applying pneumoperitoneum with precision in patients with reduced preoperative reserve.

Phrenic nerve crush, on the evidence reported above, can be genuinely irreversible in a way that is not predictable in advance [47,48]. A method whose failure mode is permanent unilateral diaphragmatic paralysis is difficult to justify as prophylaxis against PAL, a complication that is itself usually self-limited and rarely fatal. This method has effectively left contemporary practice; it is included here mainly to mark the outer bound of denervation.

Catheter-based anesthetic block is the only method with genuine postoperative titratability, since the infusion can be adjusted or stopped in response to the clinical course [13,16,17]. That advantage is bought at the cost of a continuously running delivery system for as long as the effect is needed, with its own risks of dislodgement and infection, and the logistic burden of ultrasound-guided catheter placement. Comparing catheter-related complication rates with the morbidity of a prolonged chest drain is clinically relevant to this decision. A study on phrenic nerve blocks involving 76 patients reported an accidental catheter dislodgement rate of 17.1% [68]. Two large series of peripheral nerve block catheters reported infection in approximately 3%, and the duration of catheter indwelling is a risk factor for local inflammation and infection [69,70]. Interscalene catheters were associated with an increased risk of infection (4.3%; *p* < 0.05) [70]. These figures do not map directly to thoracic phrenic nerve catheters, for which no comparably sized safety series exists, but they provide the best available external benchmark for the risk profile. Against this, a prolonged chest drain carries cumulative pain, atelectasis, empyema risk, and each additional drainage day independently adds to infection probability [62]. In the three phrenic catheter series reviewed here, air-leak cessation was achieved within a mean of 3.0 days, suggesting short catheter dwell time and correspondingly limited cumulative risk.

Phrenic nerve infiltration removes the catheter and its risks, and the one trial available shows a genuine effect on hemidiaphragmatic position, re-expansion, pain, and PAL rate [19]. Its principal limitation is temporal. Ropivacaine infiltration at the concentration and volume used in that trial is commonly reported to act for a few hours, on the order of 2 to 6 h for the infiltration technique, occasionally longer with higher concentrations or continuous delivery, but nowhere near the 78.5 ± 28.4 days reported above for BTX-A [71,72]. The biological window for pleural repair is measured in days to weeks, so a single infiltration is well suited to covering the first few hours of maximal leak volume and IPP swing, but not the window during which fibrin fixation and mesothelial repair actually take place.

Cryoneurolysis produces a genuinely reversible block without the equipment burden of a catheter, but its depth and duration are fixed at the moment of freezing and cannot subsequently be titrated [14,15]. Pan et al. reported a positive drainage-time result [14], and Meng et al. reported a null result for overall PAL rate, with a positive effect confined to the high-risk subgroup [15]. This discordance suggests the method’s benefit may depend on baseline risk in a way that has not yet been formally modeled, which limits how confidently it can be recommended outside the specific populations these two trials enrolled. The fixed duration of cryoneurolysis leaves unresolved the management of patients whose air leak persists beyond the phrenic nerve’s regenerative window. Histological data on peripheral nerve cryotherapy indicate that axonotmesis-level injury permits organized axonal regrowth, with functional recovery typically beginning at six to eight weeks [73,74,75]. This window exceeds the usual clinical course of PAL, so most patients will not encounter this scenario. For the minority who do, no published study has reported the proportion affected, described a specific management approach, or prospectively evaluated a sequential intervention strategy.

Resource dimension is also worth considering. Three of the six methods, pneumoperitoneum, phrenic nerve infiltration, and BTX-A, need no specialized equipment, which widens their potential availability beyond major centers. Cryoneurolysis requires a cryoprobe, and catheter-based block requires a continuously running infusion system for the whole treatment period, with the added consumables and monitoring burden that implies. Phrenicotripsy formally needs no equipment either, but it was excluded on safety grounds, not accessibility. On resource availability alone, then, BTX-A holds no obvious advantage over pneumoperitoneum or single-shot infiltration; its potential advantage rests entirely on its pharmacological profile, specifically the combination of controllable reversibility and duration of action.

### 4.4. Interpreting the Pharmacological Case for Botulinum Toxin Type A

BTX-A is a 150 kDa neurotoxin produced by Clostridium botulinum, whose light chain cleaves the SNAP-25 protein required for acetylcholine exocytosis [49,50]. The resulting paralysis is flaccid rather than destructive. The axon itself is preserved, and function returns through axonal sprouting, which is the pharmacological reason the effect is fully reversible rather than merely temporary in an unpredictable way [50]. This mechanism is the basis for treating BTX-A as the one method combining reversibility with a duration long enough to matter. Lebeda et al. modeled the time course mathematically, showing that functional impairment develops 12–72 h after injection, peaks by day 7–10, and plateaus for one to two months [76]. Current pharmaceutical formulations show a clinical onset of 6.7 ± 5 days and a duration of 78.5 ± 28.4 days [72]. This kinetic profile bears directly on the temporal mismatch identified above for perineural infiltration. An onset within the first postoperative week, sustained for one to three months, spans essentially the entire period during which excess negative IPP and diaphragmatic loading persist, not merely its first few hours. Among the methods compared here, this is the only pharmacological profile whose duration matches the biological repair window rather than falling short of it.

However, this argument has certain limitations. First, the mouse phrenic-nerve hemidiaphragm assay used to standardize BTX-A potency is chosen chiefly for its reproducibility: a single, easily quantified twitch response under controlled conditions [77]. It shows that the diaphragm responds predictably to the toxin in a standardized preparation. It does not establish the dose–response relationship in a human hemidiaphragm working under load. Kaya et al.’s experiment remains the entire direct evidence base for this specific indication: a single, well-designed, randomized pilot study with an objective radiographic outcome, but conducted in one species, at one dose, in a lower-lobectomy model, with six animals per arm [20].

Diffusion beyond the injected muscle is dose- and concentration-dependent, and low-volume, high-concentration injection minimizes it [78]. This carries a practical implication that the myoclonus case reports establishing BTX-A’s general safety do not address [21,79]. Confining an injection to a defined hemidiaphragm, without spreading to the contralateral side or to the pericardium, the phrenic nerve trunk, or the costal insertions, would require a dosing and injection protocol developed specifically for this anatomical target, most plausibly under intraoperative direct vision or image guidance, rather than dosing extrapolated from movement-disorder indications, where both the therapeutic goal and the target anatomy differ.

One further observation bears on the fact that more than two decades have passed since the Kaya et al. pilot was published, and to our knowledge no attempt at its in vivo replication has appeared in the literature. Possible explanations include the narrow specialization of the topic, limited commercial incentive to develop a new indication for an off-patent drug, or practical difficulty in mounting the experiment. Whichever applies, two decades without a replication attempt is itself a further limitation of the evidence base, and points to a direction of research that has gone unpursued so far.

### 4.5. Safety, Patient Selection, and the Translational Gap

A further physiological argument bears on the functional safety of BTX-A specifically, and it cuts in a more qualified direction than the myoclonus case reports alone suggest. In patients with intact lungs, unilateral diaphragmatic paralysis reduces FEV_1_ and FVC by approximately 25% owing to a restrictive shift in lung volumes, as documented in studies of interscalene brachial plexus block [80]. After lobectomy with an established RPS, elevation of the diaphragmatic dome displaces it into the residual air space itself, which contains no ventilated parenchyma, so the expected functional loss should be considerably smaller. This hypothesis requires experimental confirmation in an in vivo study.

Low FEV_1_, low diffusing capacity, and CT-confirmed emphysema amplify the PAL risk already conferred by major resection, so the patients with the strongest indication for prophylactic diaphragm management are, almost by definition, the same patients with the least respiratory reserve to absorb any transient reduction in ventilatory capacity, whatever its expected magnitude. A 25% reduction in a patient with borderline preoperative FEV_1_ is not the same clinical event as an identical percentage reduction in a patient with normal spirometry, even if the RPS-related mitigation described above holds exactly as hypothesized. This tension does not argue against the method; it argues that respiratory-reserve stratification needs to be a prespecified safety endpoint in any subsequent study, alongside diaphragmatic elevation and RPS resolution. The lowest-FEV_1_ patients may need to be studied last, once the geometric-mitigation hypothesis has been confirmed in patients with more reserve to spare.

Whether diaphragmatic intervention precipitates postoperative respiratory failure in borderline-reserve patients is currently unknown, a distinction with practical consequences for how conservatively the indication is applied. The 25% FEV_1_ reduction associated with unilateral diaphragmatic paralysis is not a fixed clinical event: its impact depends on preoperative baseline. A patient whose predicted postoperative FEV_1_ is already near 1.0 L after lobectomy is close to the threshold for dyspnoea at rest; adding a transient reduction may precipitate ventilatory decompensation requiring non-invasive or invasive respiratory support. The partial mitigation expected from diaphragmatic elevation into the residual space remains hypothetical and has not been confirmed in any postoperative population.

Risk is not uniform across the population considered for diaphragm intervention. A patient with preserved preoperative FEV_1_ who develops a large apical RPS after upper lobectomy presents a more favorable profile: high anatomical indication with adequate reserve to absorb transient functional loss. A patient with borderline FEV_1_ and extensive emphysema presents the opposite profile, one in which the anatomical argument is weaker and the functional risk is higher. Any prospective study must incorporate prespecified FEV_1_ and DLCO thresholds below which diaphragm intervention is withheld, treating these as primary safety endpoints. The lowest-reserve patients should be enrolled last, once the geometric-mitigation hypothesis has been confirmed in higher-reserve populations.

Two distinct questions follow from the geometric-mitigation hypothesis, namely whether basal diaphragmatic elevation reaches apical RPS at all and whether it risks redistributing mechanical strain toward the apex during the process. For the geometric mechanism, diaphragmatic elevation reduces total ipsilateral hemithorax volume. Because the pleural space functions as a closed, fluid-coupled cavity, this volume reduction is transmitted throughout, with pleural fluid serving as the mechanical coupling medium [35]. D’Angelo et al. demonstrated directly that diaphragmatic inactivation alters the vertical distribution of intrapleural pressure across the full height of the pleural cavity [36], confirming that changes in diaphragmatic position have cavity-wide mechanical consequences, not purely basal ones. An elevated diaphragm therefore reduces the volume available for residual space throughout the cavity, including the apex, regardless of the diaphragm’s own basal position within the thorax. Clinical evidence consistent with whole-cavity RPS reduction exists for multiple intervention types. Carboni et al. confirmed fluoroscopically that phrenic nerve block produced airspace reduction after lung resection [16]. Trabalza Marinucci et al. demonstrated a significant rise in hemidiaphragmatic position and significantly better overall lung re-expansion in patients undergoing lobectomy or anatomical segmentectomy at various sites [19], better re-expansion across the whole cavity is the expected consequence of the volume-coupling mechanism described above. For pneumoperitoneum, Okur et al. recorded substantially fewer cases of RPS (1 versus 8 in controls), and Korasidis et al. achieved obliteration of space in all 39 patients with established RPS after major resection [18,25]. Neither study specifically reported apical versus basal RPS distribution. Furthermore, none of these studies reported pulmonary parenchymal complications caused by diaphragmatic elevation. The theoretical risk for phrenic paralysis is further mitigated by the mechanism itself. Filipovic et al. mapped cyclic stretch throughout the postpneumonectomy murine lung [11], and Ysasi et al. showed that unilateral diaphragmatic paralysis after pneumonectomy reduces the mechanical stimulus that normally drives compensatory lung growth [12]. Both findings are consistent with diaphragmatic paralysis reducing parenchymal cyclic deformation, not amplifying it. For pneumoperitoneum, the diaphragm is elevated but continues to contract; it does so from a higher resting position, which reduces the ipsilateral inspiratory pressure swing and thus the peak cyclic stretch on the remaining parenchyma. This is a more specific gap than a blanket absence of evidence: no trial has stratified outcomes by RPS anatomical location, and no study has directly measured apical wall stress during any of the interventions. A further and more specific gap concerns the sole supporting experiment, which used a lower-lobectomy model in which the resulting RPS is basal and directly apposed to the injected muscle [20], whereas the highest-risk PAL population after upper and right-sided lobectomy develops an apical space at the greatest possible distance from the diaphragm within the same hemithorax. Whether hemidiaphragmatic elevation delivers a clinically meaningful reduction in apical wall stress, or whether its benefit is concentrated basally in a way that leaves the highest-risk apical defects relatively unprotected, has not been tested in either the animal model or any clinical series. This distinction is important for upper-lobe resections where PAL risk is highest and the residual space is predominantly apical, and it represents the specific measurement needed to close this question.

These risk profiles also suggest a basis for matching patients to methods. Patients whose risk is predominantly anatomical or geometric, for instance after upper lobectomy or where hemithorax volume is mismatched to the volume of lobe being removed, are the more plausible candidates for diaphragm interventions, since their problem is one of space rather than tissue quality [5,43,44]. Patients with COPD and marked emphysema, where parenchymal fragility rather than geometry is the leading mechanism, may be better served by staple-line reinforcement and defect sealing [62,63]. Pleural adhesions sit awkwardly across this divide. They are an independent PAL risk factor in their own right [54] and plausibly act through both mechanisms at once, damaging parenchyma when divided while also distorting pleural-cavity geometry, which is a reasonable rationale for a combined approach in this specific subgroup. Testing this kind of risk-based stratification, including how often patients fall cleanly into one category rather than needing a combined approach, would be a meaningful step toward personalized PAL prevention, though it remains, at this stage, a proposal rather than a tested model.

Beyond this patient-level matching, the choice of resection extent itself offers a further preventive lever at the planning stage. For eligible patients, anatomical segmentectomy produces a smaller spatial deficit than lobectomy and therefore a smaller RPS, with correspondingly lower mechanical loading at the resection margin. Two randomized trials reported relevant findings. JCOG0802 initially reported a significant overall survival advantage for segmentectomy at 5 years, but the updated 10-year analysis no longer confirmed this benefit [81,82]. CALGB140503 confirmed non-inferiority of sublobar resection for disease-free survival, but an absolute increase in local recurrence of 5% was observed across the two trials, and neither demonstrated a significant group-level advantage in postoperative pulmonary function [82,83]. As Lim E. has pointed out, the gap between trial design and clinical application lies precisely in this distinction between group-averaged functional decline and individual parenchymal preservation, a gap that must be acknowledged when interpreting the trials’ findings [84]. The absence of a measurable functional difference at group level does not negate the biomechanical rationale described here, which concerns individual-volume preservation rather than averaged functional decline. For patients who meet these oncological eligibility criteria and who simultaneously carry the anatomical PAL risk factors (right-sided or upper-lobe location, large hemithorax-to-lobe volume mismatch), segmentectomy remains an upstream PAL-prevention strategy accessible at the level of operative planning, but the decision must incorporate the recurrence trade-off and the expectation of individual functional benefit.

### 4.6. Existing Prediction Models for PAL and the Missing Mechanistic Link

Several clinically useful prediction models have been developed to stratify the risk of PAL after pulmonary resection, and these tools have important applications in patient counseling, selection for prophylactic interventions, and standardization of inclusion criteria for clinical trials.

The IPAL developed by Rivera et al. using the French national Epithor database (*n* = 24,113) includes nine variables: gender, body mass index, dyspnea score, presence of pleural adhesions, lobectomy or segmentectomy, bilobectomy, bulla resection, pulmonary volume reduction, and location on upper lobe. The model demonstrated a C-index of 0.71 in the derivation cohort and 0.69 upon external validation in 6813 patients [29]. A more recent comparison of three widely used PAL prediction models (Epithor score, Gilbert score, and Prolonged Air Leak Score) in a VATS lobectomy cohort of 534 patients found that the Epithor score showed the highest discriminatory ability (AUC = 0.735), while Prolonged Air Leak Score provided superior calibration across the clinically relevant threshold range [85].

The Brunelli scoring system, developed on 658 patients undergoing lobectomy, stratifies risk based on four variables: age > 65 years (1 point), pleural adhesions (1 point), FEV_1_ < 80% (1.5 points), and BMI < 25.5 kg/m^2^ (2 points). This model grouped patients into four risk classes with incremental PAL risk and was validated in an independent cohort of 233 patients [86]. The authors emphasized that such scores may assist in identifying high-risk patients who might benefit from prophylactic measures such as sealants or buttressed staple lines [87].

More recent work has explored advanced modeling approaches, including a nomogram for robot-assisted thoracic surgery (RATS) based on 1185 patients, which demonstrated moderate discrimination (C-statistic 0.67) and identified male sex and reduced FEV_1_ as key predictors, while also revealing a nonlinear risk increase with age beyond 70 years [88]. Despite their clinical utility, all existing models share a common limitation: they do not incorporate direct measurement of RPS volume or diaphragmatic function as predictive variables. Yet, as we have argued throughout this review, the mechanical conditions within the RPS—excess negative intrapleural pressure, loss of pleural apposition, and cyclical diaphragmatic loading are the very mechanisms that sustain fistula patency. The fact that established risk factors such as upper lobe location, right-sided surgery, and major resection independently predict both RPS and PAL is consistent with RPS serving as the shared mechanistic pathway that translates these predisposing factors into persistent air leak. We propose that future iterations of PAL prediction tools should incorporate volumetric assessment of the residual pleural space, for example, using the Collins method or CT-based quantification as demonstrated by Vita et al. [5], alongside measures of diaphragmatic elevation or intrapleural pressure dynamics as independent variables. Digital chest drainage systems that enable quantitative measurement of air-leak flow and intrapleural pressure may further enhance prediction and guide active pleural management tailored to individual patients. Such refinements would not only improve risk stratification but also provide a mechanistic rationale for selecting diaphragm-directed interventions over or alongside defect-directed measures.

#### Limitations

This review has several limitations that bear on the interpretation of its conclusions. It is a narrative rather than a systematic review, which carries an inherent degree of subjectivity in the selection and synthesis of sources. Although the literature search covered major databases using a broad range of search terms, no formal risk-of-bias assessment was performed for the included studies, a standard limitation of reviews of this type. A narrative design also does not systematically search gray literature or unpublished data, so this review cannot exclude publication bias toward positive results in the primary studies it draws on, an issue that likely applies unevenly across the methods compared, since some have several published trials while some methods (Phrenic nerve infiltration and BTX-A) have exactly one.

This review draws on evidence of fundamentally heterogeneous design, including classic physiological experiments in animal models, pilot in vivo interventional studies, randomized controlled trials, prospective and retrospective cohort studies, and clinical case reports. These sources differ substantially in their evidentiary weight, and mechanistic claims based solely on animal models or isolated clinical cases should not be equated with clinically validated fact. Several of the key studies cited here are individually small, which may inflate the apparent size of the treatment effect relative to what a different control population would show, and should be weighted accordingly. A number of the positions taken in this review are working hypotheses rather than established facts. We have tried to present them explicitly as such, as a conceptual framework requiring direct experimental testing rather than a set of settled conclusions.

Finally, this review includes literature from various regions, and the clinical studies included here differ in the definition of PAL applied (five days under the ESTS/STS consensus versus seven days in earlier work); however, definitional heterogeneity in the literature is not specific to the non-English-language sources included here. A December 2025 GRADE-based consensus from an 18-surgeon international task force identifies the absence of a single standardized PAL definition as an unresolved, field-wide problem, and separately notes that its own search, in common with most such efforts, was confined to English-language studies, a restriction the authors themselves flag as a potential source of excluded relevant research [89]. The definitional split within the regional literature cited here follows the same line that divides the English-language literature rather than a distinct national one. In addition, the differences also manifest in the surgical approach and in the composition of the operated populations. These differences limit direct comparison of quantitative outcomes, such as PAL rates or drainage duration, across studies, and call for caution in generalizing the findings summarized here to all categories of thoracic surgical patients.

## 5. Conclusions

Each of the diaphragm interventions reviewed here carries a fundamental limitation that keeps it from being optimal, and none has been tested against the others in a head-to-head trial. Chemical denervation of the diaphragm with botulinum toxin type A is, in principle, free of the irreversibility and equipment constraints that limit the other five, since it can potentially combine single intraoperative administration, predictable reversibility, and independence from permanent equipment. That principle is currently supported by a single pilot animal study. In brief, they are larger and dose-ranging, physiologically stratified by respiratory reserve, and targeted at the upper-lobe population that carries the greatest clinical burden of PAL. These strategies are also not necessarily competitors to defect-directed measures such as sealants and staple-line reinforcement. Pairing the two methods is an unstudied but plausible direction, particularly for patients who carry risk factors for both problems at once.

## Figures and Tables

**Table 1:** Overview of the incidence and related risk factors of RPS and PAL across studies.

Year	Author	Sample Size	Population	Outcome	Incidence, %	Risk Factors
2007	Misthos et al. [23]	966	Lobectomy, segmentectomy, wedge resection	RPS	9.5	Upper lobectomy, malignancy, apical location, right-sided surgery
2011	Rivera et al. [29]	24,113	All lung resections	PAL (>7 days)	6.9	Male sex, low BMI, high dyspnea score, presence of pleural adhesions, lobectomy or segmentectomy, bilobectomy, bulla resection, pulmonary volume reduction, location in the upper lobe
2017	Kim et al. [28]	1060	All lung resections	PAL	18.7	Major resection, ventilatory leak above 9.5%
2018	Jiang et al. [32]	80	Superior lobectomies	RPS	36.25	Total pleural adhesions, FEV_1_ < 1.85 L
2019	Pischik et al. [27]	319	Lobectomy, segmentectomy, wedge resection	PAL	14.7	Low BMI, hypoproteinemia, staple line length, pleural adhesions
2022	Dezube et al. [1]	2384	Lobectomy, segmentectomy, wedge resection	PAL	5.4	Male sex, older age, lobectomy or thoracotomy approach
2023	Amore et al. [33]	895	Lobectomy	PAL	8.2	Male sex, body mass index, reduced FEV_1_, pleural adhesions
2025	Ma et al. [4]	110	VATS resection	PAL	26.4	COPD, pleural adhesions
2025	Vita et al. [5]	492	Uniportal VATS lobectomy	RPS and PAL	66.1 and 9.8	Right-sided surgery, active smoking, low BMI, RPS and PAL in a bidirectional relationship
2025	Rocha Jr et al. [26]	135	Non-pneumonectomy resection due to infectious lung disease	RPS (POD 4)	58	Infectious pleuropulmonary complications

PAL, prolonged air leak. RPS, residual pleural space. POD, postoperative day. BMI, body mass index. COPD, chronic obstructive pulmonary disease. FEV_1_, forced expiratory volume in 1 s. VATS, video-assisted thoracoscopic surgery. Incidence is the proportion of the study population with the listed outcome.

**Table 2:** Comparative characteristics of diaphragm interventions for reducing the residual pleural space.

Method	Site of Action	Reversibility	Postoperative Controllability	Additional Equipment
Pneumoperitoneum [18,25,45,46,51]	Peritoneal cavity	Gradual	Partial	Not required
Phrenic nerve crush [47,48]	Phrenic nerve	May be irreversible	None	Not required
Catheter-based anesthetic block [13,16,17]	Perineural space	Complete	High	Infusion pump, catheter
Phrenic nerve infiltration [19]	Perineural fat	Complete	None	Not required
Cryoneurolysis [14,15]	Phrenic nerve	Complete	Fixed at the time of application	Cryoprobe
Chemical denervation [20]	Myoneural junction, diaphragmatic muscle	Complete	Dose-dependent	Not required

These methods have not been compared head-to-head. Entries reflect properties reported in separate studies of each method individually, not a controlled comparison.

## Data Availability

No new data were created or analyzed in this study. Data sharing does not apply to this article.

## Abbreviations

The following abbreviations are used in this manuscript:

AATS	American Association for Thoracic Surgery
BMI	Body mass index
BTX-A	Botulinum toxin type A
COPD	Chronic obstructive pulmonary disease
CT	Computed tomography
ESTS	European Society of Thoracic Surgeons
FEV_1_	Forced expiratory volume in 1 s
FVC	Forced vital capacity
GTSC	General Thoracic Surgery Club
IAP	Intra-abdominal pressure
IPAL	Index of Prolonged Air Leak
IPP	Intrapleural pressure
PAL	Prolonged air leak
POD	Postoperative day
RATS	Robot-assisted thoracic surgery
RPS	Residual pleural space
STS	Society of Thoracic Surgeons
VATS	Video-assisted thoracoscopic surgery
